# Thermo-Mechanical Performance of Epoxy Hybrid System Based on Carbon Nanotubes and Graphene Nanoparticles

**DOI:** 10.3390/nano13172427

**Published:** 2023-08-26

**Authors:** Liberata Guadagno, Carlo Naddeo, Andrea Sorrentino, Marialuigia Raimondo

**Affiliations:** 1 Department of Industrial Engineering, University of Salerno, Via Giovanni Paolo II, 132, 84084 Fisciano, Italy; lguadagno@unisa.it (L.G.); cnaddeo@unisa.it (C.N.); 2 Institute for Polymers, Composites, and Biomaterials (IPCB-CNR), Via Previati n. 1/E, 23900 Lecco, Italy; andrea.sorrentino@cnr.it

**Keywords:** hybrid nanocomposites, graphene nanosheets, carbon nanotubes, mechanical properties, thermal properties, surface analysis, tunneling atomic force microscopy (TUNA)

## Abstract

This study focuses on epoxy hybrid systems prepared by incorporating multi-wall carbon nanotubes (MWCNTs) and graphene nanosheets (GNs) at two fixed filler amounts: below (0.1 wt%) and above (0.5 wt%), with varying MWCNT:GN mix ratios. The hybrid epoxy systems exhibited remarkable electrical performance, attributed to the π–π bond interactions between the multi-wall carbon nanotubes and the graphene layers dispersed in the epoxy resin matrix. The material’s properties were characterized through dynamic mechanical and thermal analyses over a wide range of temperatures. In addition to excellent electrical properties, the formulated hybrid systems demonstrated high mechanical performance and thermal stability. Notably, the glass transition temperature of the samples reached 255 °C, and high storage modulus values at elevated temperatures were observed. The hybrid systems also displayed thermal stability up to 360 °C in air. By comparing the mechanical and electrical performance, the formulation can be optimized in terms of the electrical percolation threshold (EPT), electrical conductivity, thermostability, and mechanical parameters. This research provides valuable insights for designing advanced epoxy-based materials with multifunctional properties.

## 1. Introduction

Carbon nanotubes and graphene nanosheets have garnered significant interest due to their potential to enhance the properties of epoxy resins [1,2,3]. These enhancements encompass mechanical strength, thermal and electrical conductivity, electrochemical sensitivity, and energy storage capacity [4,5,6,7,8,9,10,11,12]. The success of these improvements relies on the structure, size, porosity, and interface of the carbon nanostructures within the epoxy matrix [4]. Furthermore, functionalization techniques can be employed to increase their compatibility and dispersion in the matrix [5]. Consequently, carbon nanostructure-reinforced epoxies find diverse applications across various industries including electronic devices, protective surfaces, adhesives, aeronautics, automotive, and marine composite materials [13,14,15,16,17]. These applications necessitate epoxies with high thermal and electrical conductivity, mechanical strength, and heat dissipation [13,14,18]. Carbon nanostructures offer distinct advantages [19,20,21] over other reinforcements such as glass or carbon fibers for epoxies including:

- Exceptional mechanical, thermal, and electrical properties such as high elastic modulus, tensile strength, thermal, and electrical conductivity.
- A high specific surface area and aspect-to-diameter ratio, which enable the formation of a three-dimensional conductive network in the matrix at low filler loads.
- A nanometric structure that can be modified to improve the compatibility and dispersion in the epoxy matrix.
- Biocompatibility, facilitating biological and medical applications.

In recent years, the combination of multi-wall carbon nanotubes (MWCNTs) and graphene nanosheets (GNs) has gained increasing attention due to the synergistic effects resulting from strong π–π interactions [6,22] and intense interfacial bonds with the polymeric matrix. The size of the reinforcing agent plays a crucial role in determining the final properties of the composite material. The combination of these two carbonaceous fillers with nanometric dimensions, each possessing unique geometrical characteristics and exceptional properties, leads to enhanced electrical, mechanical, and thermal properties in epoxy composites [6]. The hybrid combination of multi-wall carbon nanotubes (MWCNTs) and graphene nanosheets (GNs) allows researchers to exploit the advantages of both nanofillers, surpassing the performance of composites with individual nano-fillers [4]. The nanoscopic proportions of MWCNTs and GNs enable a high contact surface with the matrix, fostering enhanced synergy between the two. However, achieving uniform dispersion of the filler becomes challenging at such dimensions. The concept of “hybrid polymeric composites”, leveraging the synergistic effects of carbonaceous fillers, represents a promising strategy to achieve overall improvements in the material properties. The synergistic effect between carbon nanotubes (CNTs) and graphene nanosheets (GNs) in epoxy composites is governed by their structural and geometric interactions. Several mechanisms have been proposed [23,24,25,26,27,28] including:

- Formation of a three-dimensional hybrid structure between CNTs and GNs, which hinders face-to-face aggregation of GNs and increases the contact surface area with the matrix.
- CNTs acting as bridges between adjacent GNs, enhancing load transfer and electrical and thermal conductivity.
- Dispersion of CNTs between GNs, creating a conductive network perpendicular to the plane of GNs, improving heat dissipation and electromagnetic shielding.

Studies investigating the thermal properties of epoxy resins reinforced with CNTs and GNs have yielded significant results:

- Cross-plane thermal conductivity of reduced graphene oxide/CNT hybrid papers increased with increasing CNT loading, reaching a maximum value of 0.1199 W m^−1^ K^−1^ for a 20 wt% paper. CNTs acted as scaffolds, restraining graphene sheets from corrugating and providing more phonon transmission channels [29].
- Epoxy composites reinforced with both graphene nanoparticles and carbon nanotubes exhibited a higher tensile strength and modulus compared to those reinforced with either graphene or carbon nanotubes alone. The synergistic effect resulted from the improved dispersion and interfacial bonding of carbon nanomaterials within the epoxy matrix [25].
- Incorporating multi-wall carbon nanotubes (MWCNTs) and graphene nanoplatelets (GNPs) significantly improved the fatigue life and crack growth resistance of epoxy composites, surpassing the performance of pure epoxy or composites reinforced with only MWCNTs or GNPs. The synergistic effect was attributed to the formation of a three-dimensional network structure that increased the fracture toughness and crack bridging ability [22].

The evaluation of the thermal and dynamic mechanical properties of epoxy composites is essential for advanced material applications where thermal stability along with good mechanical performance are prime requirements [30]. Both CNTs and GNs can enhance the thermal and mechanical-dynamic properties of epoxy composites, depending on their concentration, dispersion, and interaction with the matrix [31,32]. Their interfacial bonding with the epoxy matrix improves the stiffness, strength, toughness, and fracture resistance by forming a reinforcing network and facilitating stress transfer. Additionally, the high thermal conductivity of CNTs and GNs aids in heat dissipation and in reducing thermal resistance at the interfaces [1,33]. Phonon coupling between the two carbon phases further contributes to enhanced thermal stability by improving the thermal conductivity and reducing the thermal expansion coefficient of the composite [34,35,36].

In this context, the present study investigated the synergistic effect of combining multi-wall carbon nanotubes (MWCNTs) and graphene nanosheets (GNs) in epoxy composites. Previous work [6] has shown the exceptional electrical performance of hybrid epoxy systems, attributed to π–π bond interactions between MWCNTs and graphene layers (GNs) dispersed in the epoxy resin. The hybrid systems were prepared with fixed filler amounts of 0.1 wt% and 0.5 wt%, both below and above the electrical percolation threshold (EPT) of single filler systems, with varying MWCNT:GN mix ratios. Notably, the synergistic effect was observed even at a low hybrid nanofiller amount of 0.1 wt%, solely through the addition of a small quantity of GNs, leading to a significant increase in electrical conductivity and a reduction in EPT.

Tunneling atomic force microscopy (TUNA) was employed to perform the electrical mapping of conductive nanodomains in the hybrid systems, providing valuable insights into the interface performance of the composites. Mechanical performance evaluations were conducted including dynamic and static regimes, along with thermal analysis to assess the thermal stability. Remarkable results were obtained, indicating that the hybrid epoxy formulations meet the structural requirements for aerospace and aeronautic applications. Particularly, the formulated hybrid samples demonstrated a glass transition temperature of 255 °C and high storage modulus values at elevated temperatures. Additionally, these hybrid samples exhibited a high thermal stability up to 360 °C. The findings confirm the significant synergistic effect at a low hybrid nanofiller content of 0.1 wt%, correlating well with the electrical data. A comprehensive comparison of the mechanical and electrical performance will facilitate the formulation optimization concerning the EPT, electrical conductivity, thermal stability, and mechanical parameters.

## 2. Materials and Methods

In this study, we employed multi-wall carbon nanotubes (MWCNTs) and graphene nanosheets (GNs) as conductive nanofillers. MWCNTs were produced via the catalytic carbon vapor deposition (CCVD) process and were obtained from Nanocyl S.A (Sambreville, Belgium). MWCNTs that exit the reactor are then purified to greater than 95% carbon to produce the 3100 grade employed for this work. More detailed information on the MWCNTs is contained in the Supplementary Materials.

The graphene nanosheets (GNs) used in this study were obtained through an intercalation/exfoliation procedure starting from natural graphite with an average diameter of 500 μm [11,15,37,38]. As a result of the preparation procedure, the GNs exhibited highly irregular shapes, leading to a wide distribution of lengths and thicknesses [38]. A detailed investigation of the geometric parameters of the graphene nanosheets to calculate their size is provided in the Supplementary Materials.

The preparation procedure of the unfilled epoxy matrix, denoted by the abbreviation EP deriving from the word “epoxy”, and hybrid nanocomposites, labeled using the acronym hybrid X% (MWCNT:GN) is reported in the Supplementary Materials. X% represents the weight percentage of the mix of the two nanofillers, MWCNTs and GNs, below (0.1 wt%) and above (0.5 wt%) the EPT for both nanofillers, respectively [37,39] and MWCNT:GN represents the different mix ratios of the two nanofillers MWCNTs and GNs (1:1; 1:2; 1:5; 2:1; 5:1) for the hybrid nanocomposites.

Table 1 provides the corresponding percentage by weight of each nanofiller present in the mix of the two nanofillers (MWCNTs and GNs) for each combination ratio.

Various experimental techniques were employed in this study to characterize the formulated materials including differential scanning calorimetry (DSC), thermogravimetric analysis (TGA), dynamic mechanical analysis (DMA), field emission scanning electron microscopy (FESEM), and tunneling atomic force microscopy (TUNA). Detailed technical information regarding these techniques is provided in the Supplementary Materials.

## 3. Results

### 3.1. Thermal Analysis

Dynamic mechanical and thermal analyses were conducted to characterize the properties of the hybrid nanocomposites as a function of temperature. Thermal analysis is crucial for assessing the performance and stability of these composite materials, particularly in applications within the aeronautical and aerospace sectors.

**Figure 1:**
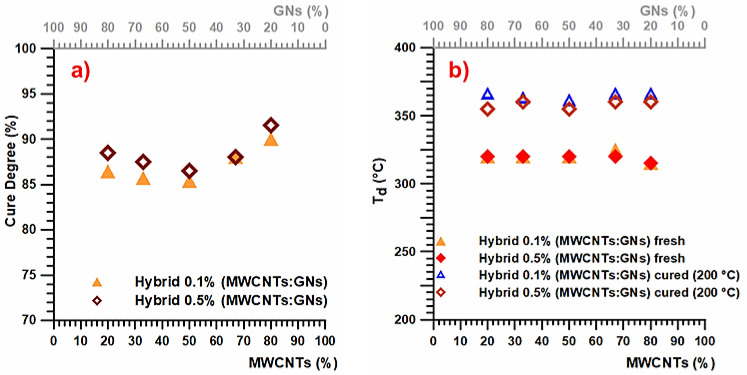
(**a**) Cure degree (%) values of the hybrid epoxy samples at 0.1 and 0.5 wt% of the carbon nanofiller mix solidified under isothermal heating conditions. (**b**) Thermodegradation temperature for the fresh (uncured) and cured (200 °C) hybrid epoxy samples at 0.1 and 0.5 wt% of the carbon nanofiller mix.

Figure 1a presents the cure degree (%) values of the epoxy samples containing 0.1 and 0.5 wt% loading of the hybrid nanofiller, namely, a mix of the two nanofillers MWCNTs and GNs (MWCNTs + GNs) (with different MWCNT:GN combination ratios) solidified under isothermal heating conditions. On the other hand, Figure 1b displays the thermodegradation temperature for both the fresh (uncured) and cured (heated at 200 °C) epoxy samples with 0.1 and 0.5 wt% loading of the hybrid nanofillers (MWCNTs + GNs) (at different MWCNT:GN combination ratios).

**Figure 2:**
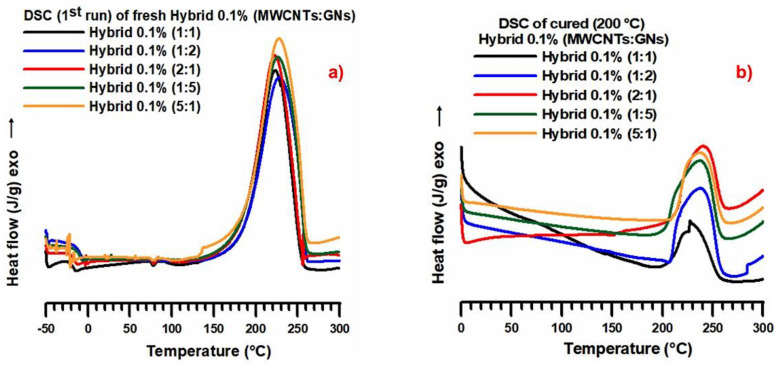
(**a**) Calorimetric curves (dynamic regime) of the fresh hybrid 0.1% (MWCNT:GN) at different mix ratios of the hybrid nanofiller. (**b**) Calorimetric curves (isothermal regime) of the cured hybrid 0.1% (MWCNT:GN) at different mix ratios of the hybrid nanofiller.

Figure 1a illustrates that under isothermal heating conditions at 200 °C, the cure degree exhibits a similar trend for both weight percentages (0.1 wt% and 0.5 wt%) of the hybrid nanofiller (MWCNTs + GNs). The highest cure degree value (~90–91 wt%) was observed in the formulation where the hybrid composition of the nanofiller had a prevalence of MWCNTs (specifically, the ratio with 80% MWCNTs), corresponding to the MWCNT:GN mix ratio of 5:1, as shown in Table 1. These results suggest a significant synergistic reinforcing effect between carbon nanotubes and graphene nanoparticles on the thermal conductivity of the composite, even with a small amount of graphene nanoparticles (20 wt%). This indicates that the hybrid nanofiller can form a three-dimensional network structure, facilitating efficient phonon transport across the composite. Figure 1b demonstrates that the different weight percentages (0.1 wt% and 0.5 wt%) of the hybrid nanofiller (MWCNTs + GNs) did not significantly impact the thermodegradation temperature (T_d_) in both the fresh (uncured) and cured samples. The curing cycle, as expected, led to an increase in T_d_ of about 40 °C. Specifically, a value of 320 °C was detected in the fresh samples, while cured samples at 200 °C exhibited a T_d_ of 360 °C. In Figure 2a, the calorimetric curves in the dynamic regime of the fresh hybrid 0.1% (MWCNT:GN) at different mix ratios of the hybrid nanofiller are displayed. Additionally, Figure 2b presents the calorimetric curves in the isothermal regime of the cured hybrid 0.1% (MWCNT:GN) at different mix ratios of the hybrid nanofiller.

From Figure 2a, it can be observed that the crosslinking peaks for the different formulations analyzed were substantially superimposable, with a peak temperature value of approximately 230 °C. Additionally, upon closer examination of the DSC traces, formulations with a hybrid nanocharge (MWCNT:CpEG) ratio of 1:1 and 2:1 exhibited a slightly narrower width at ½ height. Furthermore, a small melting peak at about 80 °C was consistently present.

In Figure 2b, the DSC traces for the hybrid epoxy samples cured up to 200 °C also showed substantially superimposable residual cross-linking peaks, with a peak temperature of about 235 °C. The DSC traces had similar amplitudes at ½ height for all of the samples investigated. Specifically, for the different epoxy formulations loaded with 0.1 wt% of the hybrid nanofiller (MWCNT + GN), the following cure degree (%) values were calculated: 85.4% for hybrid 0.1% (1:1), 85.7% for hybrid 0.1% (1:2), 86.4% for hybrid 0.1% (1:5), 88% for hybrid 0.1% (2:1), and 90% for hybrid 0.1% (5:1). Notably, even with a low amount of nanoparticles (0.1 wt%), the hybrid nanofiller concentration demonstrated a significant synergy, resulting in an increased cure degree for the hybrid epoxy samples compared to their single nanofiller counterparts, which had a higher percentage by weight (0.5 wt%) corresponding to the percentage above the EPT [6,9,39,40]. For instance, the sample EP 0.5% CNT exhibited a cure degree of 89% [41], while the sample EP 0.5% GN shows a cure degree of 86% [11]. In contrast, the unfilled resin EP exhibited a cure degree of 93% [41]. This result is particularly important as it shows that even with the lowest weight percentage (0.1 wt%) of the nanofiller mixture, it is possible to obtain hybrid formulations with cure degrees meeting the requirements imposed by the aeronautical industries, with values close to or even exceeding those exhibited by binary formulations based on a single nanofiller, loaded with the higher weight percentage of 0.5 wt%. This result is in line with the synergistic effect observed for the same low amount (0.1 wt%) of the hybrid nanofiller, resulting in an increase in the electrical conductivity of several orders of magnitude and a lowering of the EPT [6]. Specifically, the addition of graphene nanosheets (GNs) to the material filled with only MWCNTs led to an increase in both electrical conductivity and cure degree for the hybrid ternary samples. For instance, the sample hybrid 0.1% (5:1), with the lowest amount of graphene nanoparticles (GNs) at 20 wt% (see Table 1), exhibited a cure degree of 90%, which was higher than the cure degrees of 89% for the sample EP 0.5% CNT and 86% for the sample EP 0.5% GN, both of which contained only one type of nanofiller and had a weight percentage greater than 0.1 wt%.

**Figure 3:**
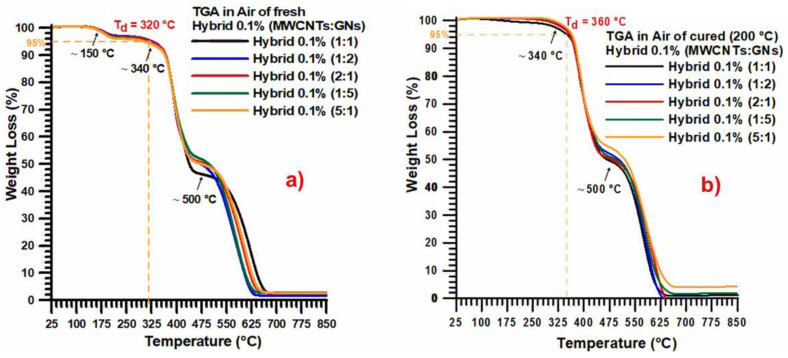
Thermogravimetric curves in air of: (**a**) the fresh (uncured) hybrid 0.1% (MWCNT:GN) at different mix ratios of the hybrid nanofiller, and (**b**) the cured hybrid 0.1% (MWCNT:GN) at different mix ratios of the hybrid nanofiller.

Figure 3 presents the thermogravimetric curves in air for the fresh (uncured) hybrid 0.1% (MWCNT:GN) at different mix ratios of the hybrid nanofiller (Figure 3a) and the cured hybrid 0.1% (MWCNT:GN) at different mix ratios of the hybrid nanofiller (Figure 3b).

The weight loss detected by the TGA curves in air for the fresh samples consisted of three steps. The initial step (with a loss of about 4%) at around 150 °C was likely to be related to the loss of free H_2_O due to the dehydration, as already found by Kandola et al. [42]. After this first slight weight loss, two main degradation steps were observable, the second step (with a weight loss of about 45%) starting at about 340 °C and the third one (with a weight loss of about 49%) starting at about 500 °C. This weight loss, characterized by two main steps, is a well-known trend for epoxy resins and is due to the fact that the oxygen is consumed primarily by gas-phase oxidation reactions during the flaming burning of the nanocomposites; oxygen hardly reaches the thermally degrading sample surface beneath the evolved gaseous products [42,43].

For all of the analyzed samples, a final residue of about 2% and a thermodegradation temperature (T_d_) of 320 °C were observed. When considering the cured samples, the weight loss detected by the TGA traces in air consisted of only two steps, as expected. The initial step (with a weight loss of about 49%) was activated at around 340 °C. The second step (with a weight loss of about 49%) was activated at about 500 °C. For all the samples investigated, a final residue of about 1–1.5% and a thermodegradation temperature (T_d_) of 360 °C were observed, which was 40 °C higher than that observed for the fresh samples (Figure 3a).

**Figure 4:**
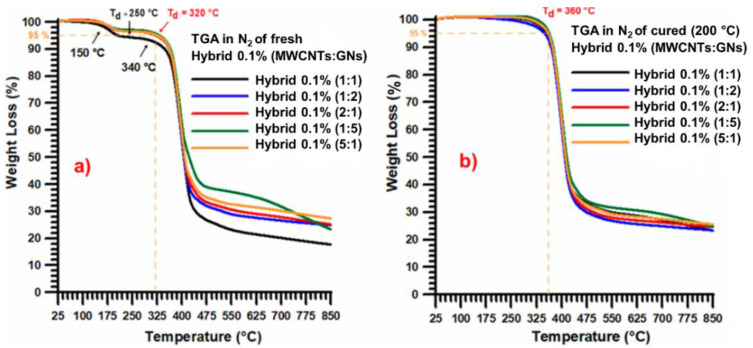
Thermogravimetric curves in nitrogen of: (**a**) the fresh (uncured) hybrid 0.1% (MWCNT:GN) at different mix ratios of the hybrid nanofiller, and (**b**) the cured hybrid 0.1% (MWCNT:GN) at different mix ratios of the hybrid nanofiller.

Figure 4 shows the thermogravimetric curves in nitrogen (N_2_) for: (a) the fresh (uncured) hybrid 0.1% (MWCNT:GN) at different mix ratios of the hybrid nanofiller, and (b) the cured hybrid 0.1% (MWCNT:GN) at different mix ratios of the hybrid nanofiller.

The weight loss detected by the TGA curves in N_2_ for the fresh samples (Figure 4a) and cured samples still consisted of three and two steps, respectively. The second thermodegradation step, with a weight loss of about 65–70% and therefore more consistent that than observed in air, was activated at around 320 °C (for the fresh samples) and 360 °C for the cured samples, as for the degradation in air, indicating that this stage is independent of the presence of oxygen. The third step (not closed at 850 °C) was activated at about 450 °C and was much slower with respect to the degradation in air, which is due to the decomposition and release of various fragments over different temperature ranges [42,43]. This behavior has been explained by the authors of Ref. [42], who combined TGA results with the FTIR spectra of the gases developed during the thermal scan in both air and nitrogen.

**Figure 5:**
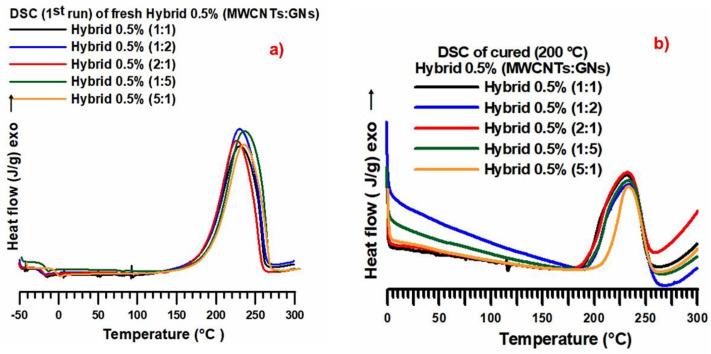
(**a**) Calorimetric curves (dynamic regime) of the fresh hybrid 0.5% (MWCNT:GN) at different mix ratios of the hybrid nanofiller. (**b**) Calorimetric curves (isothermal regime) of the cured hybrid 0.5% (MWCNT:GN) at different mix ratios of the hybrid nanofiller.

Figure 5a displays the calorimetric curves (dynamic regime) of the fresh hybrid 0.5% (MWCNT:GN) at different mix ratios of the hybrid nanofiller, while Figure 5b shows the calorimetric curves (isothermal regime) of the cured hybrid 0.5% (MWCNT:GN) at different mix ratios of the hybrid nanofiller. From Figure 5a, it can be observed that the crosslinking peaks were substantially superimposable for the different formulations analyzed (the value of the peak temperature was about 235–240 °C). The width at ½ height of the different formulations was identical, and a small melting peak at about 80 °C was also present. Similarly, from Figure 5b, the cross-linking peaks were substantially superimposable for the different formulations analyzed (the value of the peak temperature was at about 235–240 °C). The width at ½ height for the formulation hybrid 0.5% (5:1) appeared to be slightly narrower. For the different epoxy formulations loaded with 0.5% of the hybrid nanofiller, the following cure degree (%) values were calculated: 86.5% for hybrid 0.5% (1:1), 87.5% for hybrid 0.5% (1:2), 88.5% for hybrid 0.5% (1:5), 88% for hybrid 0.5% (2:1), and 91.5% for hybrid 0.5% (5:1). Comparing the cure degree values with the epoxy nanocomposites loaded with 0.5 wt% of the hybrid nanofiller at various mix ratios of MWCNT:GN, it can be observed that they were all above 86%, reaching 91.5% for the hybrid sample with the mix ratio (MWCNT:GN) of 5:1. The 5:1 mix ratio allowed us to obtain the highest cure degree of 91.5%, even in the case of the nanocomposite loaded with 0.1 wt% of the hybrid nanofiller. This result confirms the relevant synergistic effect at a low hybrid nanofiller amount, namely 0.1 wt%, finding a perfect correlation with the electrical data.

**Figure 6:**
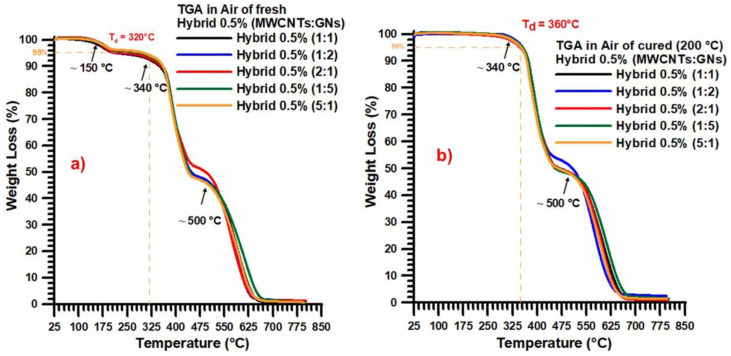
Thermogravimetric curves in air of: (**a**) the fresh (uncured) hybrid 0.5% (MWCNT:GN) at different mix ratios of the hybrid nanofiller, and (**b**) the cured hybrid 0.5% (MWCNT:GN) at different mix ratios of the hybrid nanofiller.

Figure 6 shows the thermogravimetric curves in air of: (a) the fresh (uncured) hybrid 0.5% (MWCNT:GN) at different mix ratios of the hybrid nanofiller and (b) the cured hybrid 0.5% (MWCNT:GN) at different mix ratios of the hybrid nanofiller. The presence of a higher quantity of nanofillers did not substantially alter either the profiles of the thermogravimetric curves or the temperature ranges corresponding to the different degradation events already observed in Figure 3. A similar result was also obtained for the thermogravimetric curves in nitrogen.

Table 2 shows the degradation temperature (T_d_) values for the different epoxy nanocomposites filled with carbon nanotubes and graphene nanoparticles. From this comparison, we can clearly observe that the degradation temperature (T_d_) values were very high for all of the nanocomposites listed in the table. This indicates that the addition of MWCNTs, GNPs, SiO_2_, GO, rGO, and GNs enhanced the thermal stability of the epoxy matrix. The remarkable result that emerges from the table concerns the hybrid nanocomposites presented by us in this work. For both the epoxy sample loaded with 0.5 wt% of the hybrid nanofiller and for the one loaded with 0.1 wt%, the same degradation temperature equal to 360 °C was observed. The degradation temperature of 360 °C, although lower than that recorded by the samples in the table, falls fully within the targets of structural applications with the advantage of obtaining a high thermal stability of the final material as well as for percentages of hybrid nanofiller lower 0.5 wt%.

### 3.2. Dynamic Mechanical Analysis

Dynamic mechanical tests were performed to study the viscoelastic behavior of the nanocomposites. In particular, the storage modulus and tan δ were evaluated for all of the developed samples. These two parameters provide a first immediate evaluation of the field of application. The storage modulus provides information on the ability of a material to store energy elastically. The loss tangent (tan δ) is a relevant parameter for dynamic thermomechanical properties as it is sensitive to all molecular motions in the polymer, and the temperature corresponding to its peak can be used as the glass transition temperature (Tg) of the composites [46].

**Figure 7:**
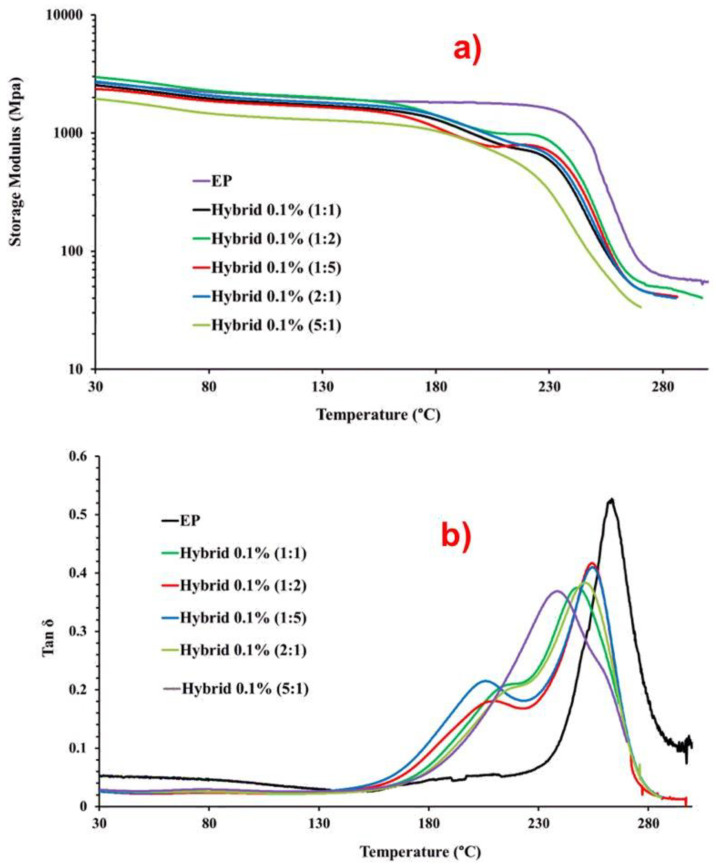
DMA results of the EP and hybrid 0.1% (MWCNT:GN) samples: (**a**) storage modulus vs. temperature, (**b**) tan δ vs. temperature.

Fiber-reinforced composite polymeric matrices exhibit a glass transition temperature (Tg), above which the material properties degrade significantly. Ensuring that the operating temperature for a polymer composite is below the glass transition is necessary to maintain acceptable mechanical stiffness and creep resistance. Thermoset materials show changes in properties such as the elastic modulus when heated through the Tg from the glassy state to the rubbery state [47]. The glass transition temperature, or a temperature well below the Tg, is often used as an upper limit for the use of polymer composites in structural applications [47]. Various methods can measure the Tg of a material such as thermal analysis techniques like differential scanning calorimetry (DSC) and dynamic mechanical analysis (DMA). DMA, sometimes referred to as dynamic thermal mechanical analysis (DMTA) or thermomechanical analysis (TMA), offers the greatest sensitivity in measuring slight transitions in polymers [47]. Highly crosslinked thermosetting resins often require DMA for Tg measurements, as methods like DSC and TMA may lack sufficient sensitivity. In this work, along with the thermal analysis previously discussed, dynamic mechanical analysis (DMA) was used to characterize the material’s properties as a function of temperature. To analyze the DMA data, the storage modulus and tan δ were plotted against temperature. Figure 7 illustrates the DMA results of the EP unfilled epoxy matrix and hybrid 0.1% (MWCNT:GN) samples cured up to 200 °C: (a) storage modulus vs. temperature; (b) tan δ vs. temperature. The temperature range shown in the two graphs of Figure 7 is from 30 °C to 300 °C.

To evaluate the effect of combining the two 1D and 2D nanofillers on the dynamic-mechanical properties of the epoxy hybrids, we also compared the trends with the unloaded epoxy matrix, as indicated by the acronym EP. Figure 7a shows that the storage modulus values for hybrid composites were slightly lower than that of the unfilled resin EP. However, for almost all samples, except for the hybrid 0.1% (5:1) sample, the modulus never dropped below 2000 MPa up to temperatures even beyond 130 °C. After an almost plateau that for the unfilled resin EP extended to 230 °C, two drop steps were observed for the hybrid nanocomposites, unlike in the profile of the EP sample, which manifested as a single drop step. The maximum value in the tan δ and the curve profile can help to understand this behavior. Table 3 presents the parameters generated by DMA, namely the Tg and storage modulus (SM), at three different temperatures (0 °C, 30 °C, and 150 °C) for the unfilled resin EP and hybrid formulations at different MWCNT:GN ratios. The data reported for the values of Tg (max. value of the main transition in the tan δ profile) show that the maximum value of Tg was detected for the unfilled sample EP. For all of the hybrid nanocomposites, lower values ranging between 248 and 255 were detected. This result seems to align with the results obtained for the curing degree of the formulated composites. In fact, the unfilled sample EP characterized by the highest curing degree (93%) manifested the highest value of Tg (248 °C), whereas the hybrid 0.1% (1:1) sample with the lower value of curing degree (85.4%) was characterized by the lowest value of Tg (148 °C). In this explanation, the sample hybrid 0.1% (5:1) was excluded because the behavior will be discussed later.

These results show that the introduction of the nanoparticles affects the crosslinking density and therefore the glass transition temperature of the samples. Another very important aspect to consider is the profile of the tan δ. Although the same curing cycle was performed for all samples including the unfilled resin EP, the introduction of the mixture of carbonaceous fillers (MWCNTs + GNs) determines two transitions in the profile of the curve (corresponding to the two steps of the module drop). This trend has been already observed for the introduction of MWCNTs alone and GNs alone [39] and results in the formation of a second phase with a lower Tg due to the greater mobility of the polymer chains and different crosslinking densities of the resin in contact with or around the nanofiller. Of course, as expected, the presence of the nanofiller interrupts the polyaddition reactions in the regions where the MWCNTs or the GNs are located. This determines discontinuities in the crosslinking density during the curing phase, which explains the presence of a second phase for hybrid composites with a lower Tg, as shown in Figure 7b.

Concerning the behavior of the hybrid 0.1% (5:1) sample, a peculiar behavior could be observed. In fact, for this sample, the main transition in tan δ was observed at the lower temperature. This sample manifested a shoulder at a higher temperature of around 260 °C, which seems to be associated with the second phase observed for all the hybrid nanocomposites. Unlike other hybrid nanocomposites, this sample’s peak at the higher temperature, which appeared as a shoulder under the whole profile, was of lower intensity. Therefore, a behavior inverse to all the others was manifested. If the interpretation of the results is correct, this behavior is indicative of the fact that a major phase in contact with the fillers is present, and therefore a more effective distribution of the filler in the hosting matrix, with MWCNTs connected through GNs. This also explains the lower storage modulus detected in Figure 7a, and the higher electrical conductivity value detected for this sample with respect to the counterpart loaded at 0.1 wt% with a single filler. In fact, the electrical conductivity of the sample loaded with 0.1 wt% of MWCNTs alone was 1.06 × 10^−13^ S/m, that of the sample loaded with 0.1% of GNs alone was 2.48 × 10^−5^, whereas the value of 2.49 × 10^−4^ S/m was observed for the nanocomposite hybrid 0.1% (5:1) [6].

**Figure 8:**
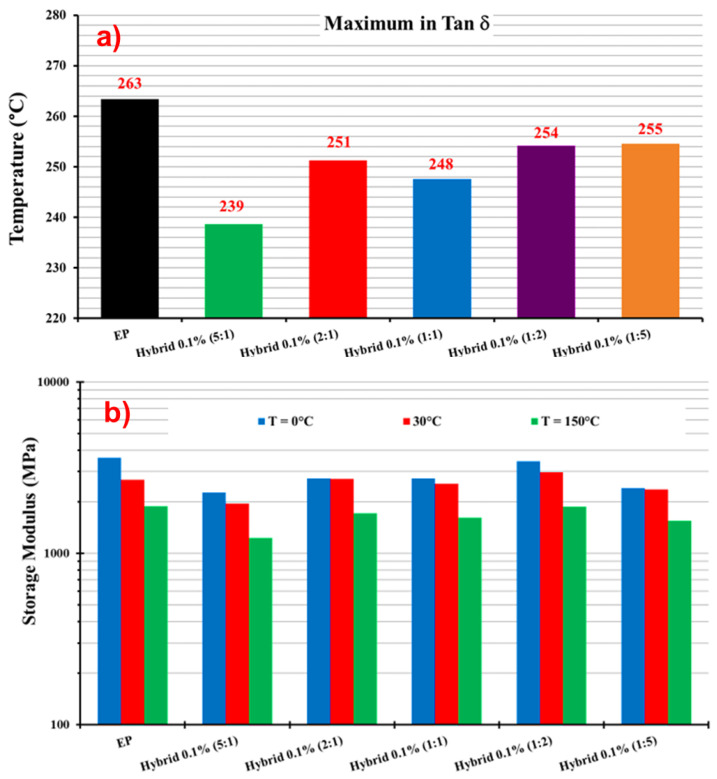
(**a**) Maximum in tan δ and (**b**) storage modulus of the EP and hybrid 0.1% (MWCNT:GN) samples at different temperatures.

The histogram in Figure 8a displays the values of maximum in Tan δ (Tg), representing the temperature at which the glass transition occurs.

Figure 8b reports the storage modulus values for the unfilled resin EP and the five hybrid formulations containing 0.1 wt% of the hybrid nanofiller (MWCNTs + GNs) at different mix ratios (MWCNT:GN) and at three different temperatures (0 °C, 30 °C, and 150 °C). At 0 °C, modulus values higher than 3000 MPa were observed for EP (3603 MPa) and hybrid 0.1% (1:2) (3438 MPa). For the other hybrid samples, the modulus values were still higher than 2000 MPa, except for hybrid 0.1% (5:1) at 1950 MPa. At 30 °C, the highest value (2977 MPa) was again recorded for hybrid 0.1% (1:2), followed by 2724 MPa for hybrid 0.1% (2:1), both higher than the value of 2690 MPa for EP. For all of the other hybrid formulations, the modulus value was still higher than 2000 MPa. At 150 °C, hybrid 0.1% (1:2) had the highest modulus value of 1874 MPa among all of the hybrids, which was slightly lower than the EP’s value of 1883 MPa. At this temperature, the hybrid 0.1% (5:1) sample had the lowest modulus value of 1231 MPa.

**Figure 9:**
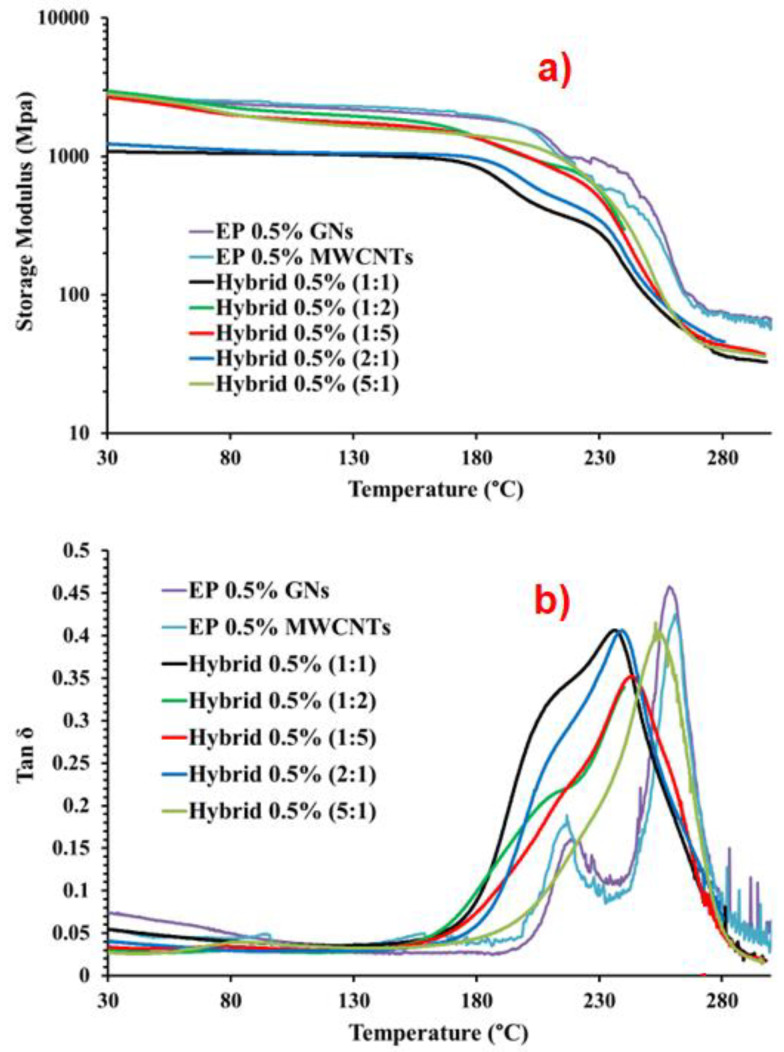
DMA results of the EP 0.5% GNs, EP 0.5% MWCNTs, and hybrid 0.5% (MWCNT:GN) samples: (**a**) storage modulus vs. temperature, (**b**) tan δ vs. temperature.

**Figure 10:**
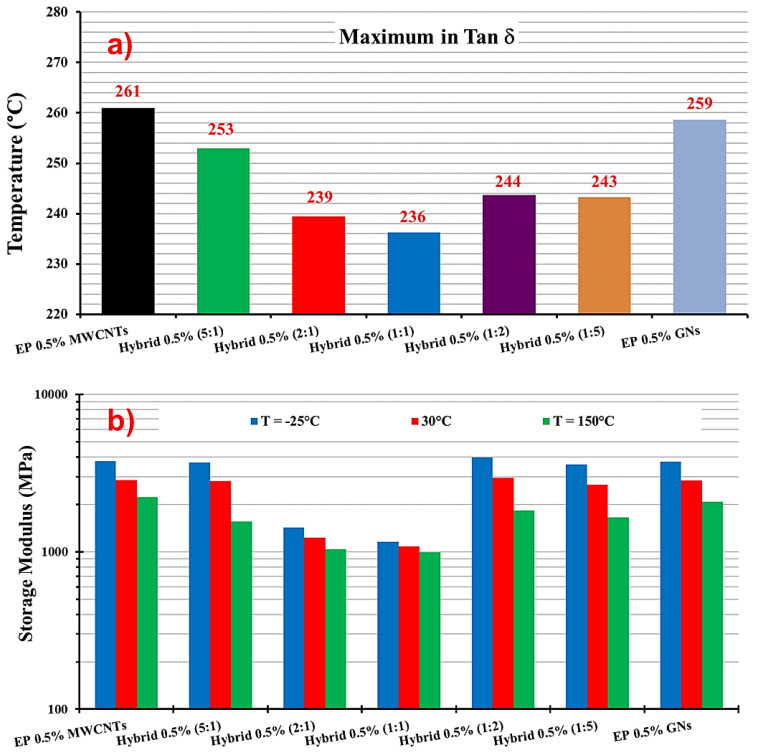
(**a**) Maximum in tan δ and (**b**) storage modulus of the EP 0.5% GNs, EP 0.5% MWCNTs, and hybrid 0.5% (MWCNT:GN) samples at different temperatures.

Figure 9 presents the DMA results of the EP 0.5% GNs, EP 0.5% MWCNTs, and hybrid 0.5% (MWCNT:GN) samples: (a) storage modulus vs. temperature; (b) tan δ vs. temperature. The temperature range shown in both graphs in Figure 9 was from 30 °C to 300 °C. Table 4 provides the values of the Tg and storage modulus (SM) for the EP 0.5% MWCNTs, EP 0.5% GNs, and hybrid 0.5% (MWCNT:GN) samples. Figure 10 shows: (a) the maximum in tan δ and (b) storage modulus of the EP 0.5% GNs, EP 0.5% MWCNTs, and hybrid 0.5% (MWCNT:GN) samples at different temperatures. In evaluating the effect of the combination of the two 1D and 2D nanofillers at 0.5 wt% (amount above the EPT) on the dynamic-mechanical properties of the epoxy hybrids, we compared the results with those obtained for the same hybrid systems but at a lower 0.1 wt% (amount below the EPT) of the two nanofiller mix. Additionally, for comparison, we included the trends of the two epoxy samples filled with 0.5 wt% of MWCNTs (EP 0.5% MWCNTs) and 0.5 wt% of GNs (EP 0.5% GNs), which corresponded to their single nanofiller counterparts. From Figure 9a, among the five hybrid samples, hybrid 0.5% (1:1) (black curve) and hybrid 0.5% (2:1) (dark turquoise curve) exhibited the lowest storage modulus values. These values were also lower than the two binary systems loaded, respectively, with 0.5% of GNs (EP 0.5% GNs) and with 0.5% of MWCNTs (EP 0.5% MWCNTs). Specifically, from Table 4 and Figure 10b, we can observe that for hybrid 0.5% (1:1), the storage modulus values were as follows: 1158 MPa at T = −25 °C, 1086 MPa at T = 30 °C, and 994 MPa at T = 150 °C. Notably, the hybrid 0.1% (1:1) sample, loaded with a smaller quantity of 0.1 wt% of hybrid nanofiller but having the same 1:1 combination ratio, had higher storage modulus values (2737 MPa at T = 0 °C, 2541 MPa at T = 30 °C, 1617 MPa at T = 150 °C) compared to the hybrid 0.5% (1:1) sample measured at the same temperatures (1130 MPa at T = 0 °C, 1086 MPa at T = 30 °C, 994 MPa at T = 150 °C).

Similarly, for hybrid 0.5% (2:1), the storage modulus values were as follows: 1428 MPa at T = −25 °C, 1232 MPa at T = 30 °C, and 1043 MPa at T = 150 °C. The hybrid 0.1% (2:1) sample also exhibited higher storage modulus values (2732 MPa at T = 0 °C, while for hybrid 0.5% (2:1), it was 1329 MPa at T = 0 °C, 2724 MPa at T = 30 °C, and 1717 MPa at T = 150 °C) compared to hybrid 0.5% (2:1). Among all of the formulations loaded with 0.5% of hybrid nanofiller, the hybrid 0.5% (1:2) sample had the highest modulus values, as follows: 3975 MPa at T = −25 °C, 2956 MPa at T = 30 °C, and 1834 MPa at T = 150 °C. Additionally, at T = −25 °C and T = 30 °C, hybrid 0.5% (1:2) had higher elastic modulus values compared to the binary systems of EP 0.5% MWCNTs (3759 MPa at T = −25 °C, 2850 MPa at T = 30 °C) and EP 0.5% GNs (3729 MPa at T = −25 °C, 2676 MPa at T = 30 °C). Table 4 and the histogram in Figure 10a illustrate the values of the maximum in tan δ (Tg) for hybrid 0.5% (MWCNT:GN) at different mix ratios of the two 1D and 2D nanofillers, and for the composites EP 0.5% MWCNTs and EP 0.5% GNs. The Tg was centered at 261 °C for EP 0.5% MWCNTs, 259 °C for EP 0.5% GNs, 253 °C for hybrid 0.5% (5:1), 239 °C for hybrid 0.5% (2:1), 236 °C for hybrid 0.5% (1:1), 244 °C for hybrid 0.5% (1:2), and 243 °C for hybrid 0.5% (1:5).

As expected, considering that a higher amount of nanofiller was dispersed in the resin, the transition at lower temperatures, corresponding to the fraction of the resin in contact with CNTs and GNs, was more intense due to a more significant amount of this phase. This confirms the correct interpretation of the dynamic mechanical spectra.

### 3.3. Morphological Analysis

**Figure 11:**
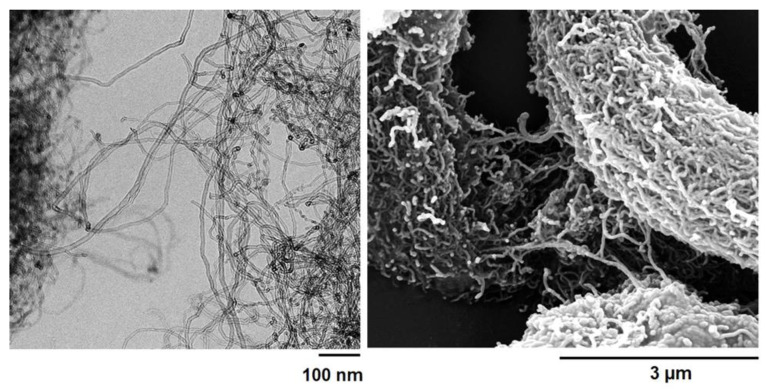
HRTEM picture (**left**) and FESEM picture (**right**) of the MWCNTs.

Figure 11 displays the high-resolution transmission electron microscopy (HRTEM) and field emission scanning electron microscopy (FESEM) images of the MWCNTs for reference.

HRTEM analysis provided the geometrical parameters shown in the Supplementary Materials. The FESEM picture shows the peculiar morphological characteristics of the multi-wall carbon nanotubes (MWCNTs), thus allowing us to better discriminate their presence between the two different types of nanofillers in the epoxy matrix.

**Figure 12:**
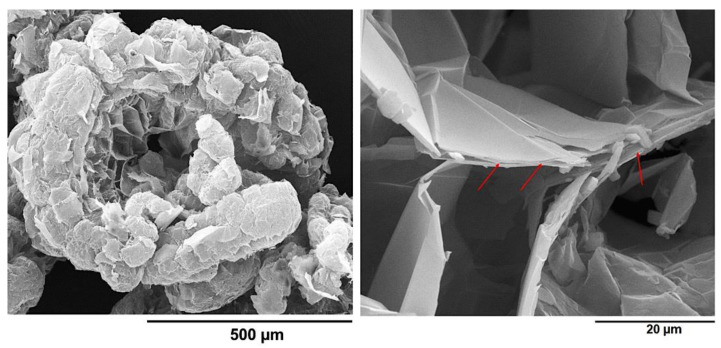
FESEM pictures of the GNs: worm-like structure (**left**) and overlapping layers of graphite (**right**).

Figure 12 displays the FESEM images of the GNs at two different magnifications. On the left, Figure 12 shows a worm-like structure of the GNs, resembling a traditional Chinese festoon or paper garland [48]. This particular morphology is characteristic of expanded graphite that has undergone a final thermal exfoliation process. At higher magnification, as shown in Figure 12 on the right, the porous structure of the GNs revealed the presence of multiple overlapping layers of graphite, some of which are indicated with red arrows.

**Figure 13:**
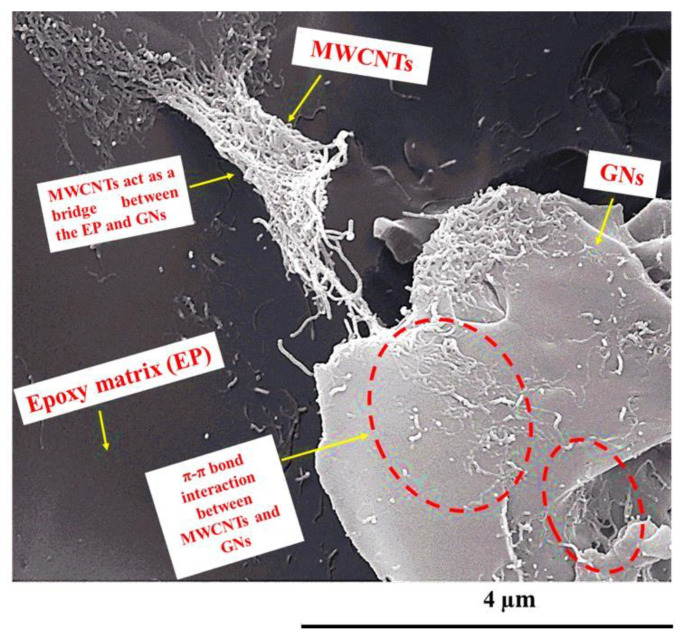
FESEM image of the hybrid 0.5% (2:1) fracture surface. Yellow arrows have been used in the Figure 13 to indicate: MWCNTs, GNs, Epoxy matrix (EP), MWCNTs acting as a bridge between the EP and GNs, and π–π bond interaction between MWCNTs and GNs (see red-dotted ellipses).

**Figure 14:**
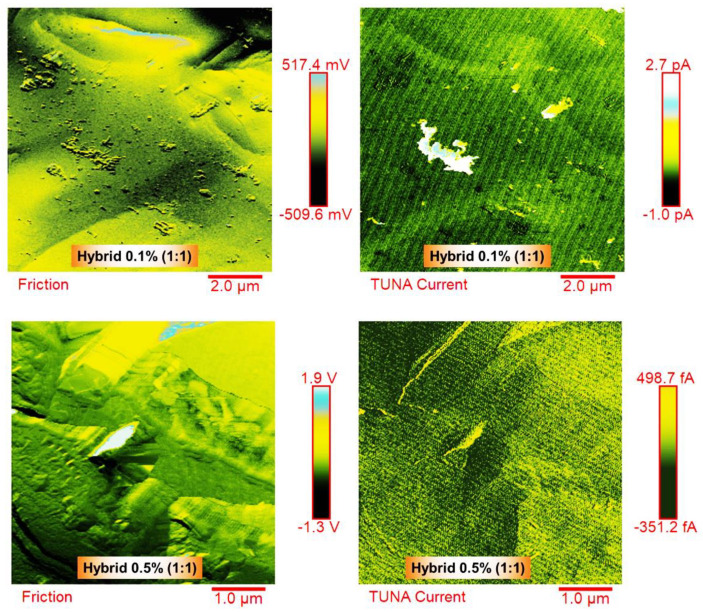
Friction and TUNA current images of the hybrid 0.1% (1:1) and hybrid 0.5% (1:1) fracture surfaces.

In order to assess the dispersion state of MWCNTs and GNs inside the epoxy matrix, examine the specific nanofiller–nanofiller and nanofiller–matrix interactions, and understand the morphological characteristics of the conductive nanoparticles that contribute to the effective interconnections responsible for the synergistic effect between 1D and 2D fillers through the π–π bond interactions established between the MWCNTs and the GNs, a morphological investigation of the hybrid nanocomposites was performed using FESEM and TUNA. Figure 13 displays a FESEM image of the fracture surface of hybrid 0.5% (2:1), while Figure 14 shows the friction and TUNA current images of the fracture surfaces of hybrid 0.1% (1:1) and hybrid 0.5% (1:1).

The oxidizing etching procedure, which effectively removed the amorphous resin, allowed for the observation of the nanofillers’ distribution within the epoxy matrix and the particular interactions responsible for the excellent electrical, thermal, and mechanical properties of the formulated hybrid samples. The FESEM image in Figure 13 is representative of the observations made in all of the hybrid nanocomposites. The EP resin appeared in a dark gray color, and the carbon nanotubes (MWCNTs) were clearly visible, emerging from the EP matrix and connecting with the overlapping graphene nanosheets (GNs). Within the red-dotted ellipses, the carbon nanotubes could be seen to form π–π bond interactions on the surface of the GNs. Moreover, upon careful examination of the image, various graphene nanolayers appeared joined together through the carbon nanotubes, which showed strong adhesion both to the GNs and the polymeric matrix, effectively acting as GN–EP bridges. The intense interactions between MWCNTs and GNs are facilitated by the particular arrangement of the carbon nanotubes, seemingly oriented at the interface with the graphene nanoplatelets, creating a highly cross-linked three-dimensional network responsible for the excellent electrical, thermal, and mechanical properties of the investigated composites. In a previous study [6], it was experimentally and computationally demonstrated that at concentrations close to the percolation threshold, the MWCNTs aggregate at the interface with the GNs, forming strong connections that enable the material to transition from an insulator to a better conductor. This transition occurs at lower concentrations, where the equilibrium between the conductor and insulator is still delicate. On the other hand, at higher MWCNT concentrations, the system already possesses well-defined conductive paths, and the addition of MWCNTs has a lower impact on the electrical properties. It is important to note that the specific orientation of the MWCNTs at the interface with the GNs was only observed in epoxy systems containing graphene nanoparticles.

The TUNA technique was employed to analyze the morphology of the conductive samples, namely hybrid 0.1% (1:1) and hybrid 0.5% (1:1), as shown in Figure 14. This analysis aimed to provide specific details about the conductive nanocharges within the host matrix and to map the local electric current values of the conductive nanodomains. Two images, friction and TUNA current, representing the same scanned area, were presented for each analyzed sample. The friction image serves as a map of the lateral flexion of the cantilever during the sample scan. This signal not only contains information about the friction between the sample and the tip, but also offers topographic details about the non-flat sample surface. This image aids in distinguishing the presence and distribution of the two types of fillers within the matrix. For both the hybrid 0.1% (1:1) and hybrid 0.5% (1:1) samples, the presence of carbon nanotubes was clearly visible on the conductive surface. In the hybrid 0.5% (1:1) sample, which contained a higher weight percentage of the hybrid nanofiller, a denser network of carbon nanotubes (MWCNTs) was detected, intertwining with each other and effectively interconnecting with the graphene nanosheets (GNs). The GNs, covering almost the entire analyzed surface, also created interfacial bonds with the host matrix. Similar to the FESEM images, the MWCNTs in the TUNA images appeared to orient themselves at the interface with the graphene nanosheets (GNs), whose edges were distinguishable. The strong π–π bond intermolecular interactions between these carbon nanostructures contributed to the formation of the highly cross-linked network, resulting in excellent electrical, thermal, and mechanical performance of the formulated hybrids. By observing the color contrast shown on the side scale bar, which ranged from darker shades for less conductive areas to lighter colors for more conductive areas, the morphological characteristics of the dispersed nanoparticles and the specific nanofiller–nanofiller and nanofiller–matrix interactions can be discriminated. The corresponding colors on the side scale bar represent the electric current values associated with the local conductive domains distributed within the matrix at the nanometric level. For the hybrid 0.1% (1:1) sample, the recorded current values ranged between −1.0 pA and 2.7 pA, while for the hybrid 0.5% (1:1) sample, the current values ranged from −351.2 fA to 498.7 fA. The TUNA technique’s ability to detect electric currents in the order of femtoamperes and picoamperes clearly indicates that the analyzed hybrid nanocomposites possessed intrinsic conductivity.

## 4. Conclusions

In this study, we performed dynamic mechanical and thermal analyses to characterize the properties of nanohybrid epoxy systems based on MWCNTs and GNs as a function of temperature. The nanohybrid systems were prepared with two total fixed filler amounts: below (0.1 wt%) and above (0.5 wt%) the electrical percolation threshold (EPT) of the single filler systems at five different MWCNT:GN mix ratios. The hybrid nanocomposites exhibited excellent electrical properties, along with high mechanical performance and thermal stability, confirming the significant synergistic effect at a low hybrid nanofiller amount (0.1 wt%). The inclusion of carbon nanotubes acted as bridges between adjacent graphene nanosheets, enhancing the load transfer and electrical properties. Remarkably, the formulated samples exhibited a high glass transition temperature of 255 °C and displayed elevated storage modulus values at high temperatures. Moreover, these hybrid composites demonstrated a remarkable thermal stability up to 360 °C in air. The observed synergistic effect can be attributed to the establishment of strong π–π interactions and intense interfacial bonds with the polymeric matrix, which was achieved with the addition of the smallest amount of graphene nanosheets (GNs) at 0.1 wt% of the nanofiller mix. The profiles of the curves tan δ vs. temperature showed two main transitions. The intensity of the first one (at a lower temperature) seemed to be directly correlated with the amount of a more mobile phase in contact with the nanofillers. Interestingly, the hybrid formulations at 0.1 wt% of nanofiller mix achieved a cure degree (DC) that fully met the requirements of the aeronautical industries, surpassing the values exhibited by the binary formulations based on a single nanofiller, even when loaded with the highest weight percentage of 0.5 wt%, corresponding to the percentage above the EPT. Specifically, for the different epoxy formulations loaded with 0.1 wt% of the hybrid nanofiller (MWCNTs + GNs), the following cure degree (%) values were calculated: 85.4% for hybrid 0.1% (1:1), 85.7% for hybrid 0.1% (1:2), 86.4% for hybrid 0.1% (1:5), 88% for hybrid 0.1% (2:1), and 90% for hybrid 0.1% (5:1). For instance, the EP 0.5% CNT sample exhibited a cure degree of 89%, while the EP 0.5% GN sample showed a cure degree of 86%. In contrast, the unfilled resin EP exhibited a cure degree of 93%. A notable result was observed for the hybrid 0.1% (1:1) sample, with the same 1:1 combination ratio but loaded with a smaller quantity of the 0.1 wt% hybrid nanofiller, showing higher storage modulus values (2737 MPa at T = 0 °C, 2541 MPa at T = 30 °C, 1617 MPa at T = 150 °C) compared to the hybrid 0.5% (1:1) sample measured at the same temperatures (1130 MPa at T = 0 °C, 1086 MPa at T = 30 °C, 994 MPa at T = 150 °C). Regarding the TUNA analysis, for the hybrid 0.1% (1:1) sample, the recorded current values ranged between −1.0 pA and 2.7 pA, while for the hybrid 0.5% (1:1) sample, the current values ranged from −351.2 fA to 498.7 fA. The TUNA technique’s ability to detect electric currents in the order of femtoamperes and picoamperes clearly indicates that the hybrid nanocomposites analyzed possess intrinsic conductivity. Looking into the future, the application of hybrid composites in the aeronautic sector holds great potential for enhancing both the structural and functional performance. The advantages include improved safety such as aircraft lightning strike protection, optimized interface properties between nanomaterials and the polymeric matrix to ensure good adhesion and load transfer as well as enhanced thermal stability and durability of hybrid composites under extreme operating conditions.

## Figures and Tables

**Table 1:** MWCNT:GN mix ratios for hybrid nanocomposites loaded with both 0.1 and 0.5 wt%.

Mix Ratios MWCNT:GN	wt% MWCNT	wt% GN
1:1	50	50
1:2	33	67
1:5	20	80
2:1	67	33
5:1	80	20

**Table 2:** Degradation temperature (T_d_) values for different epoxy nanocomposites filled with carbon nanotubes and graphene nanoparticles.

Nanocomposite	Degradation Temperature (T_d_)	Ref.
Epoxy resin filled with 0.5 wt% multi-wall carbon nanotubes (MWCNTs) and 0.5 wt% graphene nanoplatelets (GNPs)	374 °C	[22]
Epoxy resin filled with 0.5 wt% silica nanoparticles (SiO_2_) and 0.5 wt% MWCNTs	390 °C	[44]
Epoxy resin filled with 0.5 wt% MWCNTs	380 °C	[45]
Epoxy resin filled with 0.5 wt% graphene oxide (GO)	385 °C	[45]
Epoxy resin filled with 0.5 wt% reduced graphene oxide (rGO)	390 °C	[45]
Epoxy resin filled with 0.5 wt% hybrid nanofiller composed of a mix of multi-wall carbon nanotubes (MWCNTs) and graphene nanosheets (GNs)	360 °C	[This paper]
Epoxy resin filled with 0.1 wt% hybrid nanofiller composed of a mix of multi-wall carbon nanotubes (MWCNTs) and graphene nanosheets (GNs)	360 °C	[This paper]

**Table 3:** Values of the Tg and SM (MPa) of the EP and hybrid 0.1% (MWCNT:GN) samples.

Sample	Tg	SM (T = 0 °C)	SM (T = 30 °C)	SM (T = 150 °C)
EP	263	3603	2690	1883
Hybrid 0.1% (5:1)	239	2259	1950	1231
Hybrid 0.1% (2:1)	251	2732	2724	1717
Hybrid 0.1% (1:1)	248	2737	2541	1617
Hybrid 0.1% (1:2)	254	3438	2977	1874
Hybrid 0.1% (1:5)	255	2400	2357	1551

**Table 4:** Values of the Tg and SM of the EP 0.5% MWCNTs, EP 0.5% GNs and hybrid 0.5% (MWCNT:GN) samples.

Sample	Tg	SM (T = −25 °C)	SM (T = 30 °C)	SM (T = 150 °C)
EP 0.5% MWCNTs	261	3759	2850	2221
Hybrid 0.5% (5:1)	253	3688	2827	1562
Hybrid 0.5% (2:1)	239	1428	1232	1043
Hybrid 0.5% (1:1)	236	1158	1086	994
Hybrid 0.5% (1:2)	244	3975	2956	1834
Hybrid 0.5% (1:5)	243	3604	2676	1657
EP 0.5% GNs	259	3729	2831	2084

## Data Availability

Not applicable.

## Supplementary Materials

The following supporting information can be downloaded at: https://www.mdpi.com/article/10.3390/nano13172427/s1, Experimental section, Carbon nanotubes, Detailed investigation of the geometric parameters of the graphene nanosheets to calculate the size of the sheets, Preparation of the epoxy samples, Methods.
