# Supplementary material for: Thermo-Mechanical Performance of Epoxy Hybrid System Based on Carbon Nanotubes and Graphene Nanoparticles
Source: Nanomaterials (Basel). 2023 Aug 26;13(17):2427. doi: 10.3390/nano13172427 (PMC10489851; doi:10.3390/nano13172427)
Supplement: Supplementary file 1 [file nanomaterials-13-02427-s001.zip › nanomaterials-2558823-supplementary.pdf]

Supplementary electronic materials

Thermo-mechanical performance of epoxy hybrid system based on carbon nanotubes and graphene nanoparticles

Liberata Guadagno [1], Carlo Naddeo [1], Andrea Sorrentino [2] and Marialuigia Raimondo [1,*]

[1] Department of Industrial Engineering, University of Salerno, Via Giovanni Paolo II, 132, 84084 Fisciano, Italy; lguadagno@unisa.it (L.G.); cnaddeo@unisa.it (C.N.)
[2] Institute for Polymers, Composites, and Biomaterials (IPCB-CNR), Via Previati n. 1/E, 23900 Lecco, Italy; andrea.sorrentino@cnr.it (A.S.)
* Correspondence: mraimondo@unisa.it (M.R.)

Experimental section

Carbon nanotubes

The MWCNTs were sourced from Nanocyl (Sambreville, Belgium) and possessed a purity of > 95 wt%, with a diameter ranging from 10 to 30 nm, lengths varying from 100 nm to a few microns, and a number of walls between 4 and 11, with a wall separation of 0.35 nm. Brunauer–Emmett–Teller method has allowed us to determine the specific surface area of MWCNTs whose value is about 250–300 m^2/g. The thermogravimetric analysis showed a carbon purity greater than 95%.

Detailed investigation of the geometric parameters of the graphene nanosheets to calculate the size of the sheets

To have a more precise estimate of the dimensions of the graphene nanolayers, i.e. to obtain a reliable parameter not dependent on a particular shape but exclusively on the exact area, the square root of the sheet area was calculated: $s = \sqrt{area}$ [38].
The sheet size distribution was quite wide and varies between 7.5 μm and 47.5 μm. However, a prevalence of frequency between 10 μm and 25 μm could be observed. This evidence confirmed that thermal expansion during the exfoliation process caused a sharp reduction in the size of the sheets initially characterized by an average diameter of 500 μm [11,15,37,38]. The GNs nanosheets in the resin consisted of small multilayer stacks of graphene that are from 1 to about 16 nm thick, with diameters ranging from sub-micrometer to hundreds of μm. The distance between graphitic stacks was approximately between 5 and 10 nm. GNs nanoparticles contained graphitic blocks composed of a number of layers between 5 and 29. GNs had an amount of carboxylated groups of about 10 wt% [37].

Preparation of the epoxy samples

The epoxy matrix, denoted by the abbreviation EP deriving from the word "epoxy", is composed of a blend of tetraglycidyl methylene dianiline (TGMDA) and 1,4-butanedioldiglycidylether (BDE) in an 80:20 wt% proportion. The curing agent used is 4,4'-diaminodiphenyl sulfone (DDS), added at a stoichiometric concentration of 55 parts by weight per hundred parts of the resin (phr) blend TGMDA+BDE. All these chemical products were sourced from Sigma-Aldrich (Milan, Italy).

The hybrid nanocomposites were prepared by homogeneously adding and dispersing both the one-dimensional (1D) and two-dimensional (2D) nanofillers in the appropriate mix ratios into the epoxy blend (TGMDA+BDE) at a temperature of 90 °C for 20 minutes, utilizing a Hielscher model UP200S (200 W, 24 kHz) ultrasound system from Hielscher Ultrasonics GmbH, Teltow, Germany. Subsequently, the temperature was increased to 120 °C, and the curing agent 4,4'-diaminodiphenyl sulfone (DDS) was added to the mixture, which was continuously stirred magnetically until complete solubilization of the crosslinking agent was achieved.

Afterward, the final liquid nanofilled formulations were degassed at approximately 100 °C under vacuum for about 1 hour to eliminate any bubbles caused by entrapped air in the mixture. Solidification of EP unfilled resin and all hybrid epoxy samples was carried out in two isothermal stages in suitable molds inside an oven: a first stage at 125 °C for 1 hour followed by a second stage at higher temperatures up to 200 °C for 3 hours. This procedure contemplates, for the first curing step, times and temperatures lower than those required for the second step. It corresponds to the common industrial conditions aimed at ensuring an optimal impregnation of the carbon fibers at lower temperatures before the resin solidification at higher temperatures takes place.

In this paper, the hybrid epoxy samples were labeled using the acronym Hybrid X% (MWCNTs:GNs), where X% represents the total weight percentages (0.1 and 0.5 wt%) of the two nanofiller mix, and (MWCNTs:GNs) indicates one of the five mix ratios (1:1; 1:2; 1:5; 2:1; 5:1) of MWCNTs and GNs, respectively. To investigate the dynamic-mechanical behavior of the epoxy hybrids loaded with 0.5 wt% (above EPT) of the nanofiller mix and evaluate the effect of the synergistic interaction between graphene nanosheets (GNs) and multi-wall carbon nanotubes (MWCNTs), a comparison was made with the tan δ and storage modulus values of MWCNTs/epoxy nanocomposites and GNs/epoxy nanocomposites, where both MWCNTs and GNs were individually dispersed at 0.5 wt% (above EPT). The epoxy nanocomposites loaded with 0.5 wt% of MWCNTs and GNs were abbreviated in the manuscript as EP 0.5% MWCNTs and EP 0.5% GNs, respectively.

Methods

Two types of thermal investigation were performed on the formulated samples, Differential Scanning Calorimetry (DSC) and Thermogravimetric Analyses (TGA). DSC analyses have been carried out by using a thermal analyser Mettler DSC 822/400 (Mettler-Toledo Columbus, OH, USA) equipped with DSC cell purged with nitrogen and chilled with liquid nitrogen for sub-ambient measurements. DSC has been employed to evaluate the samples' cure degree (DC), assuming that exothermic heat developed during the curing process is proportional to the extent of the curing reactions. The DC can be determined from the total heat of reaction (ΔH_T) of the curing reactions and the residual heat of reaction (ΔH_{Res}) of the partially cured resin according to Equation 1.

$$DC = \frac{\Delta H_T - \Delta H_{Res}}{\Delta H_T} \times 100 \quad (1)$$

The total heat of reaction (ΔH_T) has been determined by performing the DSC analysis on the liquid uncured samples, scanning about 7.0 mg of the sample by a heating run at 10 °C/min from -50 °C to 300 °C, while the ΔH_{Res} was determined from the measurements performed on the oven hardened samples, by scanning the polymerized materials at 10 °C/min from 0 °C to 300 °C.

The analysis of the uncured samples was carried out by means of a dynamic heating program that contemplates three stages in the temperature range between -50 °C and 300 °C, namely: a) a first run from -50 °C up to 300 °C with a scan rate of 10 °C/min, b) a second run from 300 °C to -50 °C with a scan rate of 50 °C/min and c) a third run from -50 °C up to 300 °C with a scan rate of 10 °C/min. The samples oven cured at 200 °C were analyzed by single heating run from 0 °C up to 300°C with a scan rate of 10°C/min.

Thermogravimetric analysis (TGA) (Mettler TGA/SDTA 851 thermobalance) was accomplished by heating the samples from 25 °C to 850 °C at a 10 °C/min heating rate under both nitrogen and air flows.

Dynamic mechanical properties of the solid samples with dimensions 2 × 10 × 35 mm^3 were performed with a dynamic mechanical thermo-analyzer (Tritec 2000 DMA -Triton Technology) by applying a variable flexural deformation in three points bending mode. The displacement amplitude was set to 0.03 mm, whereas the measurements were performed at the frequency of 1 Hz. The range of temperature analyzed in the DMA

graphs of Figures 7,9 was from 30 °C to 300 °C at the scanning rate of 3 °C/min. In the graph of Figure 8b), and Table 3 values of the storage modulus were measured for the samples EP and Hybrid 0.1% (MWCNTs:GNs) also at T = ° C. In the graph of Figure 10b), and Table 4 values of the storage modulus were measured for the samples EP 0.5% GNs, EP 0.5% MWCNTs and Hybrid 0.5% (MWCNTs:GNs) also at T = -25 °C.

High-Resolution Transmission Electron Microscopy - HRTEM (Jeol 2010 LaBa6 microscope operating at an acceleration voltage of 200 kV) was used to investigate the morphology of the MWCNTs.
MWCNTs were dispersed (in ethanol) by ultrasonic waves for 30 min. The obtained suspension was dropped on a copper grid (holey carbon).
Field Emission Scanning Electron Microscopy – FESEM (mod. LEO 1525, Carl Zeiss SMT AG, Oberkochen, Germany) was used to investigate the morphology of the MWCNTs, GNs, and the fracture surface of the nanocomposite Hybrid 0.5% (2:1). All the samples were placed on a carbon tab previously stuck to an aluminum stub (Agar Scientific, Stansted, UK) and were covered with a 250 Å-thick gold film using a sputter coater (Agar mod. 108 A).

The morphological characterization of the fracture surfaces of the nanocomposites Hybrid 0.1% (1:1) and Hybrid 0.5% (1:1) was carried out by Tunneling Atomic Force Microscopy (TUNA) technique. Nanofilled sample sections were cut from solid samples by a sledge microtome. These slices were etched before the observation by FESEM and TUNA to discover the carbon nanofillers because they are deprived of the surrounding resin layer which is consumed by the oxidizing solution. The etching reagent was prepared by stirring 1.0 g potassium permanganate in a solution mixture of 95 mL sulfuric acid (95–97%) and 48 mL orthophosphoric acid (85%). The filled resins were immersed into the fresh etching reagent at room temperature and held under agitation for 36 h. Subsequent washings were done using a cold mixture of two parts by volume of concentrated sulfuric acid and seven parts of water. Afterward, the samples were washed again with 30% aqueous hydrogen peroxide to remove any manganese dioxide. The samples were finally washed with distilled water and kept under vacuum for 5 days before being subjected to morphological analysis.

Information on topography and local electrical current of the hybrid nanocomposites was obtained by the TUNA technique operating in contact mode and using platinum-coated probes with nominal spring constants of 35 N m−1 and an electrically conductive tip of 20 nm.

The TUNA module measures ultra-low currents (<1 pA) ranging from 80 fA to 120 pA circulating through the conductive tip to the investigated samples kept at a fixed DC bias. In this work, we used a DC sample bias from 1 V to 2 V.

A linear current amplifier with a 60-120 fA range detects the resulting current passing through the samples. In this way, the sample's topography and current are measured at the same time, activating a direct correlation of a sample location with its electrical properties.

It is worth noting that notably sensitive current measurements are allowed due to the noise level of the TUNA module commonly of 50 fA. The highest resolution current mapping of the nanocomposites was obtained with the current sensitivity of the TUNA module which selects the gain referring to the output voltage of the TUNA sensor set to 1pA/V, corresponding to the gain of 10^{12}, scan rate of 0.500 Hz s^{-1}, number of pixels in X and Y (samples/line) set to 512.

The detected areas of the analyzed samples are representative of the entire hybrid nanocomposites because, in order to obtain electrical measurements at the nanoscale level with indisputable repeatability and reproducibility, a cantilever with a sharp tip has scanned different areas over a sample surface so that each TUNA image reported in the manuscript was captured after verifying that the electrical response was the same at least five various scanned points.

It is worth noting that, generally, it is not enough that the tip is in contact with a conductive material but electrical contacts to the ground ensured by silver paste are also essential for the current to flow.

Thus, a current signal is obtained only if the tip during the sample contact constitutes a part of a closed electrical circuit. In this work, the electrical characterization at the nanoscale level was carried out without grounding the samples.

The TUNA results show that, even if the analyzed samples are not grounded, it is possible to detect electric current values that irrefutably prove the intrinsic electrical conductivity of the formulated nanocomposites.

The TUNA images were analyzed using the Bruker software Nanoscope Analysis 1.80 (Build R1.126200).
